# Examining How Oral Nicotine Pouches Are Trending on TikTok: A Qualitative Descriptive Study

**DOI:** 10.2196/73032

**Published:** 2025-11-14

**Authors:** Ashmeet Mand, Karlee Fonteyne, Laura Struik

**Affiliations:** 1 University of British Columbia Okanagan Kelowna, BC Canada

**Keywords:** qualitative research, oral nicotine pouch, ONP, TikTok, Zyn, youth, social media

## Abstract

**Background:**

Oral nicotine pouches (ONPs) have become a popular alternative nicotine product in recent years, especially among youth. This is concerning given the substantial health risks associated with nicotine use at an early age. Social media channels that are popular among youth (eg, TikTok) are being used to promote ONPs. Understanding the ways that individuals communicate about ONPs on popular social media, like TikTok, is critical for informing public health efforts aimed at protecting youth from the harms of nicotine.

**Objective:**

This study aimed to explore how ONPs (specifically Zyns) are represented on TikTok.

**Methods:**

Through a qualitative descriptive design, TikTok videos were collected and analyzed. A total of 250 TikTok videos were screened under the hashtags #zyn, #zyns, and #nicotinepouch, with 132 included for final analysis. Analysis was guided by the Braun and Clarke thematic analysis approach. Collaborative coding ensured reliability and supported the identification of key themes in the videos.

**Results:**

The following five themes emerged: (1) the Zyn movement (58/132, 43.9%), which relays how ONPs have become a popular culture trend; (2) “boy heaven” (28/132, 21.2%), which showcases the dominance of male use; (3) unintended negative consequences (18/132, 13.6%), which reveals how negative side effects are downplayed; (4) product design: life does not need to stop (13/132, 9.8%), which highlights how ONPs enable nicotine consumption anywhere at any time; and (5) physical benefits: “It’s like IcyHot for your mouth” (6/132, 4.5%), which brings forward positive sensory experiences with ONP use. Overall, the content heavily condoned ONP use and normalized it, with male individuals disproportionately represented as the primary users of ONPs.

**Conclusions:**

This study reveals TikTok’s influence in shaping the perceptions of ONPs as trendy and relatively harmless, especially among youth. The findings highlight how these products can quickly become embedded in youth culture through popular social media channels. The findings also underscore the need for more restrictions on the content that is so readily available to youth.

## Introduction

### Background

In recent years, novel forms of nicotine consumption have surged in popularity. A relatively new addition to the landscape of nicotine products is oral nicotine pouches (ONPs), which has grabbed the attention of youth. A recent scoping review based in the United States estimated that adolescent lifetime use of ONPs was 3.5% to 4.1% and current use was 1.5% to 2.0% [1]. It is likely that these numbers will climb within this demographic as ONP use continues to infiltrate the lives of today’s youth [1,2].

ONPs are slim microfiber pouches filled with white, finely granular nicotine salts [3-5]. ONPs typically contain between 2 mg and 10 mg of nicotine per pouch, subject to country-specific regulations; however, some pouches have been reported to contain up to 50 mg of nicotine per individual pouch [2-7]. These pouches may come in a myriad of flavors, such as berry, citrus, and mint, making them more enticing to users [8,9]. Similar to traditional snus (a smokeless tobacco-based product), ONPs are inserted between the gum line and the cheek. However, unlike snus, ONPs are touted as “tobacco free” and are frequently marketed as a healthier option for nicotine users [1,8,10]. There is not enough evidence to date to definitively determine whether ONPs are less harmful than their electronic cigarette (e-cigarette) counterparts [1,2,11]. That said, ample research has emphasized the negative health implications of acute and chronic nicotine exposure, particularly among youth, including neurodevelopmental and cardiovascular effects, as well as a relationship with mental health issues [12-14]. Despite the negative health implications of nicotine use, several studies have found that users of ONPs perceived them to be less harmful compared with other available nicotine products [1,2,10,15].

Brands (eg, market leader Zyn, a subsidiary of Philip Morris International) [7] selling and marketing ONPs have pushed the idea that these products are discreet, convenient, and trendy, and use buzzwords like “freedom,” “flavor,” and “innovation” [8,15,16]. Furthermore, these companies have capitalized on the reach and power of social media to market ONPs, particularly targeting the youth demographic [8]. Research has shown that social media plays a role in influencing trends among youth, including the use of nicotine products, by increasing their social acceptability and desirability and lowering their harm perception [17-20]. Unlike traditional advertising channels, social media platforms facilitate peer-to-peer sharing, amplify exposure via algorithms, and create participatory cultures where behaviors can quickly become normalized. This makes them a particularly influential force in shaping youth perceptions of nicotine products.

One of the most popular social media platforms used among youth, where ONPs are being promoted, is TikTok [17,21,22]. TikTok was first introduced in 2017 and has become one of the fastest-growing social media platforms [23]. The hallmark feature of TikTok is the ability to create short videos with different effects (eg, overlaid music or text, filters) that can then be posted and shared via hashtags. As of 2024, there were an estimated 2 billion TikTok users worldwide. According to Pew Research Center [24], 63% of teenagers (aged 14-17 years) in the United States use TikTok, and more than half of these users report using it daily. TikTok’s format of highly visual, short-form content encourages the rapid dissemination of trends and narratives, making it a key hub for examining how ONPs are portrayed and engaged with in real time.

### This Study

Two recent content analyses examined the presence and promotion of ONPs on the TikTok platform [21,22]. Both Donalson et al [21] and Zenone et al [22] highlighted the normalization of ONP use, the reliance on humor, and the minimization of associated risks, noting a lack of cessation-related content. While these content analyses provided timely and valuable insights into the promotional strategies surrounding ONPs and the presence of ONP content on TikTok, they captured a limited picture of the meaning embedded in user-generated posts. Given that engagement with nicotine-related social media content has been linked to an increased likelihood of product use [20], it is critical to further examine how ONPs are represented on TikTok. Therefore, this study used a thematic analysis to move toward an in-depth understanding of how users express their experiences and perspectives on ONPs. By doing so, it offers additional insights into the ways ONP use is constructed, communicated, and normalized in digital spaces, extending the current literature and informing future prevention, policy, and cessation efforts.

## Methods

### Study Design

Given the lack of research in this emerging area, we used qualitative description, which is well suited for exploring and describing phenomena where research is lacking [25,26]. This study focused on surfacing the meaning of ONP use conveyed through TikTok videos without excessive theoretical abstraction. This methodological approach ultimately led to actionable findings for public health efforts.

This study involved 3 coauthors: a senior researcher, an emerging researcher, and a youth coresearcher. This authorship enabled blending of the expertise of the senior and emerging researchers with the lived experience and cultural immersion of the youth coresearcher as it related to TikTok and nicotine use trends within the youth demographics. The entire study process, from conceptualization to knowledge translation, was a collaborative endeavor.

### Ethical Consideration

TikTok has multiple options for users to restrict their content through privacy settings. For example, users can choose between a public and private account and can also select “who can view” each video. Public accounts can set each TikTok video to be viewed by friends, everyone, or only themselves. Therefore, ethical clearance was deemed unnecessary, as TikTok videos posted from public accounts with no viewing restrictions indicate that the creators intended their content to be widely accessible. This approach is in line with previous studies examining TikTok videos [27].

Only posts that were publicly accessible and downloadable were included in the thematic analysis, with the assumption that creators had no expectation of privacy and that their videos were part of the public domain. To minimize the potential for harm, all usernames and profile images were removed, and direct quotes were paraphrased when possible or used only when necessary to illustrate a theme. Paraphrasing was applied when the exact wording was not essential to the meaning, which reduced the likelihood of reverse identification through search engines. However, when specific phrasing, slang, or tone was central to understanding the theme, direct quotes were retained. This approach balanced the need to preserve interpretive fidelity with the imperative to protect user anonymity.

We also acknowledge the ethical implications of studying youth behavior on social media, particularly given the sensitivity of nicotine use among minors. Our analysis was strictly observational, with no interaction or engagement with users. The TikTok account used for data collection was created solely for research purposes, with the age set to 15 years to ensure the algorithm displayed content representative of what midadolescent users might encounter.

### Data Collection

TikTok videos containing the hashtags #zyn, #zyns, and #nicotinepouch were analyzed. These hashtags were selected because, at the time of data collection (May 13, 2024), they were the most frequently used and most frequently engaged tags related to ONPs, collectively encompassing approximately 45,000 posts. Content under these hashtags included personal accounts of ONP use, as well as discussions from significant others about a partner’s use. A total of 250 TikTok videos were selected from a combined pool of content under the 3 hashtags. This combined approach was used because many TikTok videos contained more than one of the selected hashtags, with some including 2 or even all 3 hashtags. Pooling avoided duplicate content and ensured the sample represented the overall range of popular ONP content on the platform. The 250-video threshold was chosen because TikTok’s algorithm organizes videos based on engagement metrics such as likes, views, and interactions; therefore, the first 250 videos represented the most popular content available at that time. From the initial pool, we excluded any TikTok videos that (1) were not in English, (2) were not downloadable, (3) did not mention ONPs or Zyns, or (4) were posted before January 2023. After applying these exclusion criteria, 132 TikTok videos remained for analysis. The number of likes, comments, and shares for each TikTok was noted and recorded on a Microsoft Excel sheet.

### Data Analysis

**Figure 1:**
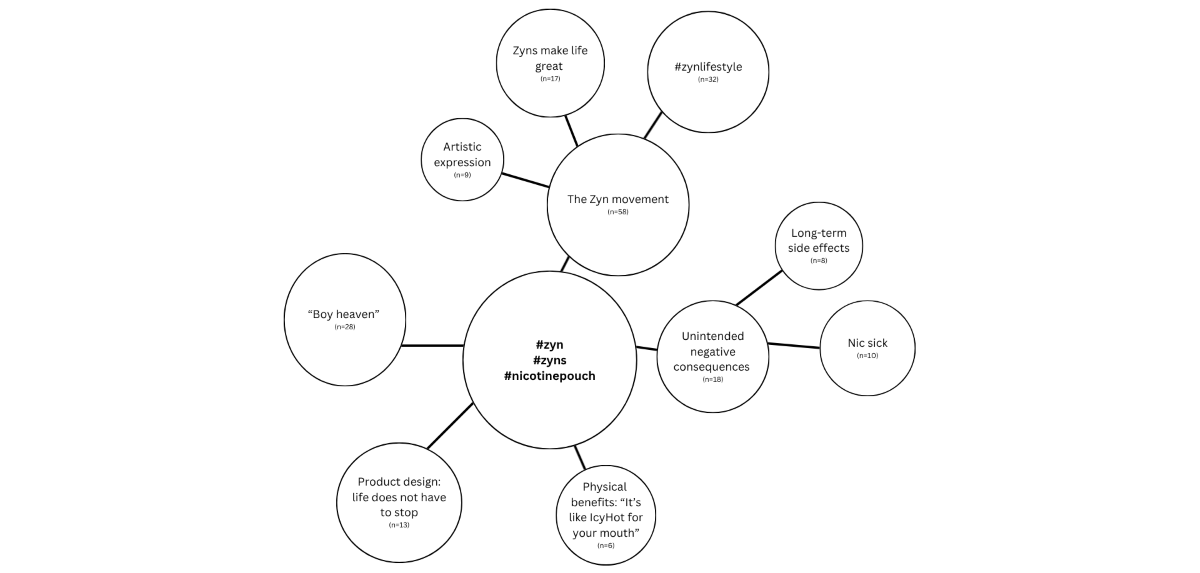
Themes identified across content posted under #zyn, #zyns, and #nicotinepouch on TikTok. The five main themes are represented by larger circles, with their size reflecting the relative prominence of the theme within the dataset. These themes include: (1) the Zyn movement, (2) "boy heaven", (3) product design: life doesn’t have to stop, (4) unintended negative consequences, and (5) physical benefit: “It’s like IcyHot for your mouth.” Subthemes are shown in smaller circles connected to their corresponding theme. Within the Zyn movement, subthemes include (a) #zynlifestyle, (b) Zyns make life great, and (c) artistic expression. Within unintended negative consequences, subthemes include (a) nic sick and (b) long-term side effects. Lines connecting shapes indicate conceptual relationships observed during coding.

The first author (AM) and the third author (LS) analyzed the compiled TikTok videos guided by the Braun and Clarke 6-step approach for thematic analysis [28]. For the first step (familiarization), we viewed the collected TikTok videos and discussed our observations and how to approach the coding process. For the second step (initial code generation), the analysis of these videos began with individually coding the first 20 TikTok videos. Each TikTok was watched at least 3 times. Then, we met and discussed our findings to ensure there was consistency within the analysis. This process was repeated with the first 50 TikTok videos to ensure consistency in the coding process. The first author (AM) then completed coding the remaining TikTok videos. It is noteworthy that the familiarity of the youth coresearcher with the lingo used on TikTok and the social trends associated with nicotine use among younger demographics was critical to the analytic process because this familiarity enabled an accurate depiction of the dominant themes. This process helped to develop a codebook that was accurately representative of the content and increased interrater reliability. During step 3 (development of themes), after all the videos were coded, AM and LS engaged in several collaborative meetings to discuss how these codes naturally group together to form themes. For example, while we had codes relating to the ability to conceal use, the ability to use ONPs anywhere, and the ability to engage in sports or military training while using them, it became evident that there was an underlying discourse that life does not need to stop consuming nicotine with ONPs. Therefore, our primary theme became “life doesn’t have to stop.” During step 4 (theme refinement), we collaboratively reviewed themes and ensured that our themes and associated subthemes were capturing the video content appropriately. For step 5 (theme naming), we built a diagram to showcase how each theme and subtheme related to each other (Figure 1). Within our findings, we indicated the number of videos associated with each theme to showcase the prominence of the themes, as well as the degree to which videos conveyed ONPs in a positive or negative way.

## Results

### Overview

The authors (AM and LS) interpreted the data to form 5 themes (two of which have subthemes), as shown in Figure 1. The authors understood that each individual TikTok varied greatly despite sharing the same hashtag, so each video was coded according to the central message the creator was trying to deliver. These TikTok videos generated a sum of 16,488,662 likes; 114,125 comments; and 1,930,115 shares. From the most common theme to the least, the themes are (1) the Zyn movement, (2) “boy heaven”, (3) product design: life does not need to stop, (4) unintended negative consequences, and (5) physical benefits: “It’s like Icyhot for your mouth.”

### The Zyn Movement

#### Overview

Creators (58/132, 43.9%) showcased their alliance with using Zyns by posting content in favor of Zyns and ONPs. This theme captured how creators framed Zyn use as empowering, exclusive, and socially desirable, often presenting participation in the “movement” as part of a distinct social identity. The Zyn movement was a common hashtag under videos where Zyns were mentioned, signaling membership in a shared identity group. Within this theme, 3 subthemes were identified: (1) #Zynlifestyle, (2) Zyns make life great, and (3) artistic expression.

#### #Zynlifestyle

This subtheme showcased how Zyns could become a part of someone’s lifestyle. A lifestyle describes a way in which a person chooses to live their life. Creators (32/132, 24.2%) posted videos portraying Zyns as a seamless part of their day, whether at work, watching sports, playing sports, or on vacation. One creator even called Zyns the “perfect ball marker” while playing golf, illustrating how the product was framed as both practical and socially embedded within leisure activities. Creators also showed Zyns being used as tools, such as a car jack and a line marker, reinforcing the portrayal of Zyns as adaptable to many contexts. Collectively, these examples depicted Zyn use as normalized and compatible with diverse lifestyles.

#### Zyns Make Life Great

This subtheme promoted the use of Zyns as something that makes life great. Creators (17/132, 12.8%) made TikTok videos expressing how great Zyns are and how they have made their lives better. For example, one creator made a 2-minute video solely expressing his love for Zyns, directly reflecting the *Zyns make life great* narrative. Another showcased a soda alongside a Zyn with the caption, “Just copped the ‘Why do I feel so good all the time? starter pack’,” implying that Zyns are part of a recipe for happiness. Similarly, a creator posted, “How are you sad in a generation where wintergreen zyns, coors banquets, and girls in 7s exist,” framing Zyns as central to maintaining a positive mood. Together, these posts position Zyn use as a simple and reliable pathway to a better life.

#### Artistic Expression

This subtheme highlighted the creativity of creators who incorporated Zyns into their TikTok videos to showcase the product’s versatility. Zyns were not only presented as a source of nicotine but also as a medium for artistic expression. Creators (9/132, 6.8%) featured Zyn cans in decorations, music, toys, and participation in TikTok trends. For example, the hashtag #zynmas appeared under videos displaying Zyn cans as Christmas tree ornaments, demonstrating seasonal creativity tied to the product. One creator made her boyfriend a cake identical to a Zyn can, while others incorporated the cans into trending TikTok formats. TikTok trends are typically short-lived periods when a particular song or dance becomes widely popular, and participating can help creators remain visible and engaged with audiences. Within this subtheme, Zyns were integrated into these trends in ways that positioned them as a creative prop rather than solely a nicotine product.

### “Boy Heaven”

Creators (28/132, 21.2%) portrayed Zyns as a source of pleasure and indulgence, often tied to male identity or relationships. The phrase “boy heaven” appeared in a video highlighting how cheap and accessible Zyns were at a festival, directly capturing this theme’s essence. Many of these videos were made by what appeared to be males or their significant others, with some partners commenting that they “just couldn’t get mad” because the Zyn “rewards” were so good. One creator assembled a care package for her military boyfriend containing multiple Zyn cans, presenting them as thoughtful and valued gifts. Captions such as “perfect boy’s night” and “just a guy lunch” paired Zyns with alcohol or energy drinks, reinforcing the idea that Zyns were central to the ideal male social experience, akin to a personal “heaven on Earth.”

### Unintended Negative Consequences

#### Overview

Creators (18/132, 13.6%) often reached out to social media to share their experiences with using ONPs. Whether it was their first day of use or consistent use, there was a lot of testimonial-style content. One user, who presented their content through a podcast on TikTok, labeled the side effects of nicotine consumption, specifically through ONPs, as “unintended negative consequences”, presenting them as mere coincidences. This phrase was a strong representation of how the harms associated with nicotine consumption are minimized, with many creators choosing to dismiss them altogether. This theme was divided into 2 subthemes: (1) nic sick and (2) long-term side effects.

#### Nic Sick

This subtheme highlighted the negative physical reactions to nicotine, particularly among first-time users of Zyns or other ONPs. Commonly referred to as “nic sick,” these symptoms include nausea, sweating, and vomiting. Creators (10/132, 7.5%) shared “storytimes” about their severe first-day experiences, often framing them with humor or dramatic storytelling. One creator, confident from prior vaping experience, tried a Zyn and reported they “felt like they won’t make it till tomorrow” and “slept in the fetal position,” illustrating the intensity of nicotine’s acute effects despite previous exposure. In some cases, the severity of symptoms led creators to describe fearing for their well-being, while still presenting these incidents as simultaneously cautionary tales and entertaining content.

#### Long-Term Side Effects

This subtheme highlighted the health issues associated with prolonged ONP use. Creators (8/132, 6%) featured content describing impacts such as gum recession, tooth decay, and, in severe cases, oral cancer, often showing visible dental changes on camera. Some posts also referenced nicotine’s potential impact on cardiac health. These videos presented ONPs, like Zyns, as capable of causing damage to the user’s physical health.

### Product Design: Life Does Not Need to Stop

Creators (13/132, 9.8%) produced videos highlighting the convenience, discreteness, and shareability of ONPs, as well as the ability to use multiple at once. Content showed individuals using Zyns while working out, at work, and during everyday activities. Athletes such as golfers, hockey players, and baseball players were seen using Zyns midgame or interacting with fans, with some videos showing fans offering Zyns and athletes accepting them, illustrating how easily the product could be distributed and shared. Other posts emphasized discreet use in restricted environments, such as soldiers using Zyns while on duty. In addition, 2 creators demonstrated using multiple pouches simultaneously, showcasing how nicotine intake can be readily increased and customized. Overall, these videos presented Zyns as a product that integrated seamlessly into both professional and leisure activities without interrupting daily routines.

### Physical Benefits: “It’s Like IcyHot for Your Mouth.”

Creators (6/132, 4.5%) produced videos promoting perceived physical and cognitive benefits of ONPs. Reported benefits included nicotine being described as a nootropic, which, in lay terms, is a work enhancer and a substance that increases mental acuity. For male individuals, it was linked to boosting testosterone and referred to as a “male enhancer.” Some posts suggested it could aid weight management, with one creator recommending its use for severe weight problems. Statements such as “I don’t think nicotine hurts anything” and “Zyn is not a sin” reflected a minimization of potential harms in favor of perceived benefits. One creator compared ONPs to the popular muscle relief topical medication IcyHot (drug class: menthol and methyl salicylate), which is known to provide significant relief. This comparison further solidified how these creators portrayed ONPs as beneficial.

## Discussion

### Principal Findings

This study revealed themes around how ONPs are experienced, perceived, and promoted among TikTok users. The implications of these findings are critical for advancing health promotion efforts so that they resonate with individuals who use these platforms, particularly youth. A primary finding is the resoundingly positive way in which ONPs, and Zyns in particular, were portrayed in TikTok videos (eg, “boy heaven,” “unintended negative consequences,” and “life doesn’t have to stop”). This echoed findings from previous works examining how nicotine products popular among youth were engaged with on TikTok, such as vaping [17-20], as well as the two previously mentioned content analyses of ONPs on TikTok [21,22]. For example, Sun et al [19] found that, of a sample of 808 TikTok videos that were collectively viewed more than 1.5 billion times, the majority (n=509, 62.9%) portrayed e-cigarette use in a positive way. Taken together, the findings not only underscore how novel nicotine products are being positively portrayed on popular youth-led social media platforms but also explain why these products have become so widely proliferated in youth cultures.

A second key finding was the way in which ONPs are not promoted as a cessation product, for which they were supposedly intended, but rather portrayed as a lifestyle product. Some videos directly referred to the use of ONPs as a movement (eg, #zynlifestyle, #zynbabwe, and #zyngang), and other videos associated the use of ONPs as something that improves everyday life (eg, “Zyns make life great; product design: life does not have to stop”). This is similar to how vaping became a lifestyle product through similar social media posts and hashtags (eg, #vapenation and #vapefam), thereby normalizing the use of e-cigarettes as a part of everyday life and positioning them as a communal experience [18,29]. In this regard, social media serves as a powerful tool for the tobacco industry in normalizing the use of their products among youth, a prime demographic to secure profits from because of the developmental propensity of youth to become addicted to their products for the long term [12,30].

Another important finding was that individuals who appeared to be male created most of the TikTok videos about ONP use, or showed ONPs being used by what appeared to be male individuals. This is reflected in our theme, “boy heaven.” This emphasized that the use of ONPs encompassed possible gender-based dynamics whereby the use of these products is portrayed as a dominantly masculine endeavor. For example, male role models, including athletes, military personnel, and influencers, were represented in these TikTok videos, which not only reinforced and promoted the use of pouches among men, but also showed that perceived “healthy men” and “masculine men” are using these products. Furthermore, while male individuals presented as the primary users of ONPs on TikTok, female individuals were presented as supporters of their male counterparts’ use, which foregrounded stereotypical gender roles playing out. For example, in many of the videos, the female partners, while they may not have agreed with the behavior, still showed support for ONP use by creating art with the tins (eg, a Zyn birthday cake for her boyfriend), sharing that they could not become angry at their partner because they received free items through the rewards and by remaining silent when their partners announced that they are running out to obtain pouches. Not only did these findings align with studies indicating that more male individuals use ONPs compared with female individuals to date [1,31,32] but they also provided a glimpse into how gender is influencing and reinforcing these trends.

### Implications

A major implication of this study’s findings was the need to address ONPs as lifestyle products, given their widespread normalization among youth as represented in the analyzed TikTok videos. Once a trend, like ONP use, becomes embedded in youth contexts and is accelerated via social media, it becomes normalized, which inherently makes such trends difficult to counter. This brings forward the need to shift focus from presenting ONP use as solely a health issue to acknowledging that it is also a lifestyle trend embedded into youth cultures. Efforts to educate youth about the harms of these products may fall flat if we only focus on this issue for its health implications. This was exemplified in the way that the negative health impacts of ONP use were downplayed or presented as a fluke, or as an “unintended” side effect of using them. Therefore, there is an urgent need to design prevention messaging that resonates with youth cultures and to empower youth to believe that they do not need to engage in ONP use to fit in or to feel good. Designing youth-driven and peer-based interventions, disseminating targeted prevention and cessation messaging via popular social media platforms, and harnessing the power of social media influencers to counter and challenge these trends are promising ways in which to positively engage youth on this issue.

There currently appears to be a gender gap when it comes to ONP use, whereby men are overwhelmingly represented as pouch users on TikTok. This is similar to how smoking and vaping began, but through clever marketing, the gender gap began to close. One can expect that the same gender gap will close shortly without due attention to how women may be particularly targeted.

Social media regarding Zyns capitalizes on the concept of social bonding among users. Users establish small communities named #zyngang, #zymbabwe, and #zynlifestyle on social media platforms, which represent a safe space for expressing their passion for ONPs (Zyns). These communities can become particularly appealing to young people as they increasingly value and develop social connections and social capital. As per the Erikson theory [33] of psychosocial development, youth aged 12 to 18 years belong in the “identity versus role confusion” stage. This stage is where youth are figuring out their identity and what their values are, ultimately driving their decision-making around where they belong. This is why Zyn communities on social media can further increase the uptake of these products among youth. Therefore, stricter policies are needed for hashtags and content. TikTok has community guidelines for products such as cannabis, which aim to block the content from being viewed by anyone who is underage; however, there is currently no policy in place for ONPs.

Furthermore, education needs to be provided to individuals who interact with youth daily, such as parents, teachers, and coaches. They should be provided with education and resources on ONPs, as well as how to engage in productive conversations with youth about ONPs. Because the product design is so slick and easily concealable, schools will need support to address its use in classrooms. In addition, health care professionals, such as pharmacists, need to be educated on the actual consequences of these products. There needs to be an increase in awareness of the negative consequences of this product, so they cannot continue to be dismissed as “unintended.”

Finally, research is needed to further understand the use of ONPs, particularly among youth. Future studies that qualitatively explore the subjective experiences among diverse ONP users are needed, as well as quantitative explorations of how social media portrayals may be linked to uptake among younger demographics. Health promotion efforts also need to be developed and tested so that more proactive protection of youth health can be achieved.

### Strengths and Limitations

A major strength of this study was that, through a thematic analysis, it surfaced the meanings underpinning ONP-related posts on the most popular social media platform used among youth, TikTok. Another strength of this study was that it was co-led by a youth coresearcher alongside an emerging researcher and a senior researcher. This is a major strength because the lead author was familiar with the platform and with the lingo and the ways in which youth cultures are represented on this platform, which facilitated rigorous theme development. Finally, this study was strengthened by the collaborative coding and theme development process that characterized data analysis.

In relation to limitations, it is important to note that this study was conducted in 2024 and that rapid shifts in social media policies (eg, the temporary ban on TikTok in the United States), as well as ONP policies (eg, US Food and Drug Administration approval of Zyn marketing), may alter the ways in which ONPs are communicated. In addition, TikTok trends are highly temporal; hashtags, sounds, and viral formats can rise and fade within days or weeks. As such, the popularity, tone, and framing of ONP-related content captured in this study reflect a specific moment in time. While future analyses conducted months or years later may identify different creators or engagement patterns, the thematic insights derived from this dataset offer enduring relevance for understanding adolescent exposure and risk messaging in social media environments. This temporal volatility reinforces the importance of continuous monitoring. In addition, most TikTok users are based in the United States, which limits Canadian representation in this study. Because only English-language content was included, our findings may not be generalizable to non–English-speaking populations. While European creators were not intentionally excluded, their representation in the dataset was low because of the English-language focus and TikTok’s algorithm prioritizing content from US-based creators.

This study also excluded content on snus, which limits the applicability of these findings to other oral-based nicotine products that may be popular and portrayed on social media. Furthermore, this content is representative of Zyn and does not include content shared in relation to other ONP brands (eg, Rogue and Zonnic), which is likely due to the higher proliferation of Zyn in the United States than other brands to date. However, this focus on Zyn may overlook other ONP brands that are popular in different regions. Finally, TikTok is a constantly growing platform, with millions of videos posted daily from all over the world. TikTok is algorithm focused, meaning it tracks the type of videos users watch to curate their experience. However, to make our findings unbiased, we created a new account on a new device with no previous data to be tracked and used. In addition, because the TikTok account used for data collection was set to a user age of 15 years, the algorithm may have prioritized content tailored to younger users, which could limit the generalizability of the findings to other age groups.

### Conclusions

In this study, we analyzed TikTok content related to ONPs, revealing how the trend has become embedded in youth cultures through positive portrayals that resonate strongly with youth. The themes identified in our analysis illustrated how marketing and peer-to-peer promotion highlighted perceived benefits of Zyn while subtly leveraging masculinity and the male identity to normalize and glamorize use. This gendered dimension of ONP promotion is both novel and concerning, underscoring a pressing public health need to address how such dynamics may shape attitudes and behaviors. Policy and prevention efforts, ideally co-designed with youth, are urgently required to counter these messages and support informed decision-making. Further research is also essential to guide interventions and monitor how emerging nicotine products continue to evolve within rapidly shifting social media landscapes.

## Data Availability

The datasets generated or analyzed during this study are available from the corresponding author upon reasonable request.

## Abbreviations

e-cigarette: electronic cigarette
ONP: oral nicotine pouch
